# Loss of PTEN expression predicts resistance to EGFR-targeted monoclonal antibodies in patients with metastatic colorectal cancer

**DOI:** 10.1038/sj.bjc.6605575

**Published:** 2010-02-16

**Authors:** C Mao, R-Y Liao, Q Chen

**Affiliations:** 1 Department of Epidemiology, School of Public Health and Tropical Medicine, Southern Medical University, Guangzhou, China

**Sir,**

Phosphatase, homologue to tensin (PTEN), is a tumour suppressor protein that regulates the PI3K/AKT signalling pathway. Loss of PTEN expression results in overactivation of the Akt pathway and confers resistance to inhibitors of the epidermal growth factor receptor (EGFR). Recently, four clinical studies have evaluated the association between PTEN expression status and response to the treatment of EGFR-targeted monoclonal antibodies (cetuximab and panitumumab) in patients with metastatic colorectal cancer (mCRC) (Frattini *et al*, 2007; Loupakis *et al*, 2009; Molinari *et al*, 2009; Sartore-Bianchi *et al*, 2009). However, results are still inconclusive partially because of the relatively small sample size, and the retrospective and not controlled nature of these studies.

To derive a more precise estimation of the relationship, we performed this meta-analysis. The sample sizes in the four studies ranged from 12 to 85. In total, PTEN immunohistochemical analysis was performed successfully on 231 primary tumours. Loss of PTEN expression was detected in 87 (38%) primary tumours. Among 231 patients analysed, 205 patients were assessable for tumour response. The objective response rate (ORR) of mCRC patients with loss of PTEN expression was 6% (5 of 81), whereas the ORR of mCRC patients with normal PTEN expression was 32% (40 of 124). Loss of PTEN expression had a negative effect on tumour response to anti-EGFR monoclonal antibodies (pooled risk ratio, 0.22; 95% confidence interval, 0.10–0.50; *P*<0.001), with no heterogeneity between studies (*P*=0.22) (Table 1). No publication bias was found by Egger's test (*t*=1.44; *P*=0.45).

In conclusion, this meta-analysis suggests that loss of PTEN expression is associated with clinical resistance to EGFR-targeted monoclonal antibodies in patients with mCRC. However, the number of studies and the number of subjects included in the meta-analysis are relatively small. Large prospective studies using standardised unbiased methods are needed to confirm our results.

## Figures and Tables

**Table 1:** Tumour response to EGFR-targeted monoclonal antibodies according to PTEN expression status

			**ORR (%)**			
**Author (year)**	**Tumours evaluated**	**PTEN loss**	**PTEN loss**	**PTEN normal**	**Pooled RR (95% CI)**	***P*_h_**
Frattini *et al* (2007)	27	11	0/11 (0)	10/16 (63)	0.22(0.10-0.50)	0.22
Loupakis *et al* (2009)	85	36	4/36 (11)	11/49 (22)		
Molinari *et al* (2009)	38	8	0/2 (0)	2/10 (20)		
Sartore-Bianchi *et al* (2009)	81	32	1/32 (3)	17/49 (35)		

Abbreviations: CI=confidence interval; ORR=objective response rate; *P*_h_=*P* value of *Q* test for heterogeneity test; PTEN=phosphatase, homologue to tensin; RR=risk ratio.
